# State HCV Incidence and Policies Related to HCV Preventive and Treatment Services for Persons Who Inject Drugs — United States, 2015–2016

**DOI:** 10.15585/mmwr.mm6618a2

**Published:** 2017-05-12

**Authors:** Cecily A. Campbell, Lauren Canary, Nicole Smith, Eyasu Teshale, A. Blythe Ryerson, John W. Ward

**Affiliations:** 1 Division of Viral Hepatitis, National Center for HIV/AIDS, Viral Hepatitis, STD, and TB Prevention, CDC.

Hepatitis C is associated with more deaths in the United States than 60 other infectious diseases reported to CDC combined. Despite curative hepatitis C virus (HCV) therapies and known preventive measures to interrupt transmission, new HCV infections have increased in recent years (*1*,*2*). Injection drug use is the primary risk factor for new HCV infections (*2*). One potential strategy to decrease the prevalence of HCV is to create and strengthen public health laws and policies aimed specifically at reducing transmission risks among persons who inject drugs. To evaluate factors affecting access to HCV preventive and treatment services, CDC assessed state laws governing access to safe injection equipment and Medicaid policies related to sobriety requirements for approval of HCV treatment for persons who inject drugs. Acute HCV incidence rates were obtained from CDC’s National Notifiable Disease Surveillance System (NNDSS). States were categorized based on analysis of laws related to access to clean needles and syringes and Medicaid HCV treatment policies associated with sobriety requirements. In 2015, HCV incidence remained high in the United States, with rates in 17 states exceeding the national average. Three states were determined to have state laws and Medicaid policies capable of comprehensively preventing and treating HCV among persons who inject drugs. Opportunities exist for states to adopt laws and policies that could help increase access to HCV preventive and treatment services reducing the number of persons at risk for HCV transmission and disease.

HCV transmission primarily occurs through percutaneous exposure to blood; thus, injection drug use is an important risk factor (*3*). HCV incidence has increased 294% nationally from 2010 to 2015 (*4*). This increase in acute cases of HCV is largely attributed to injection drug use (*2*). Access to safe injection equipment for persons who inject drugs can prevent HCV infection (*3*), and HCV therapy can cure >90% of infected persons, thereby reducing the risk for HCV-associated mortality and transmission of HCV to others. State laws and policies can enhance or limit access to HCV prevention and treatment services, particularly for persons who inject drugs (*5*). For example, recent studies have shown that states policies can reduce deaths associated with drug overdose (*6*).

Incidence of HCV per 100,000 population was calculated based on acute cases of HCV reported electronically by each state and the District of Columbia (hereafter referred to as states) to NNDSS in 2015 and U.S. Census data. Existing state laws in all states related to access to clean needles and syringes by persons who inject drugs were reviewed using the legal database WestlawNext. Once the relevant laws were identified, the legal findings were corroborated with findings from the Syringe Distribution Laws data set on LawAtlas.* The state laws were then provided to health departments in all states for review of accuracy of interpretation.

Three types of laws related to access to clean needles and syringes were researched: 1) authorization of syringe exchange programs; 2) the scope of drug paraphernalia laws; and 3) retail sale of needles and syringes. Two independent analysts qualitatively assessed the laws based on the presence of five elements in each type of law, and the potential impact of these elements on access to clean needles and syringes, in a method similar to other legal analyses (*7*). The elements assessed included whether state laws explicitly 1) authorize syringe exchange statewide or in selected jurisdictions; 2) exempt needles or syringes from the definition of drug paraphernalia; 3) decriminalize the possession and distribution of syringes or needles for participants of a legally authorized syringe exchange program; 4) allow for a person to avoid criminal prosecution for possession of drug paraphernalia by disclosing to an arresting officer that they possess a needle or sharp object; and 5) allow for the retail sale of syringes without a prescription to persons who inject drugs. The analysts grouped the laws into five categories (most comprehensive, more comprehensive, moderately comprehensive, less comprehensive, and least comprehensive) based on the presence or absence of the five elements.

Data on Medicaid fee-for-service HCV treatment policies were collected from a report developed by the Center for Health Law and Policy Innovation of Harvard Law School and the National Viral Hepatitis Roundtable on Medicaid access to hepatitis C treatment (*8*). The Medicaid treatment policies were provided to the states for review of accuracy and updated, as needed. Based on the length of required sobriety from alcohol and/or drugs provided by the states, CDC characterized the state’s Medicaid treatment policy as either restrictive or permissive depending on the presence or absence of a sobriety requirement. For this analysis, any required period of sobriety, including requirements that a person could not have any evidence of active injection drug use, was considered a barrier and, therefore, restrictive. Screening and counseling requirements were not considered barriers, and were therefore categorized as permissive, given that those services did not necessarily require a referral or postponement of treatment. A state policy that did not require any period of sobriety was also categorized as permissive.

In 2015, the national reported acute HCV incidence rate was 0.8 per 100,000 persons, representing 2,436 new infections reported from 40 state health departments; with adjustment to account for underascertainment and underreporting, the reported number of cases is estimated to represent 33,900 new HCV infections (*4*). Incidence in 17 states exceeded the national average, including seven states with rates at least twice the national average (Figure 1).

**FIGURE 1:**
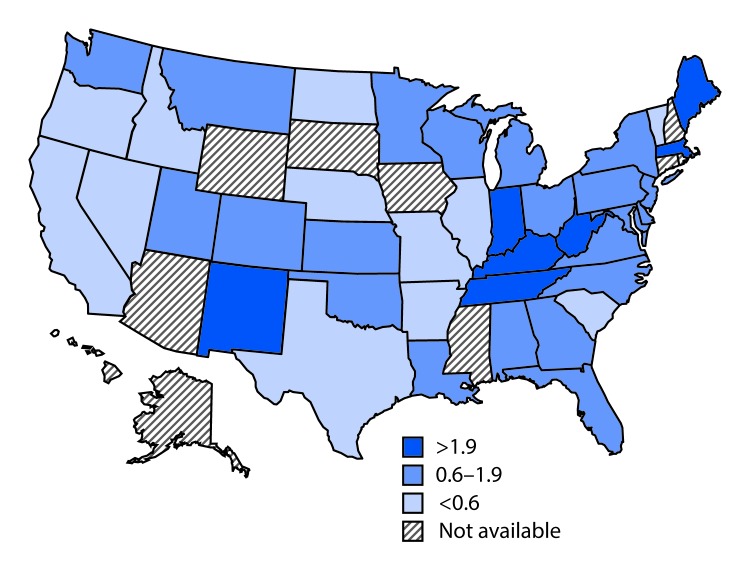
Acute hepatitis C virus infection incidence rate ratios* — United States,^†^ 2015 * The national rate (0.8 per 100,000 population) is the denominator. ^†^ Seven states have rates at least twice the national average: Indiana, Kentucky, Maine, Massachusetts, New Mexico, Tennessee, and West Virginia. Ten states have rates above the national average (but not twice the national average): Alabama, Montana, New Jersey, North Carolina, Ohio, Oklahoma, Pennsylvania, Utah, Washington, and Wisconsin.

Eighteen states had laws that were categorized as least comprehensive related to the prevention of HCV transmission among persons who inject drugs. In particular, these 18 states had no laws authorizing a syringe exchange program, decriminalizing possession and distribution of syringes and needles, or allowing the retail sale of syringes without a prescription. Three states (Maine, Nevada, and Utah) had the most comprehensive laws related to prevention; each state had laws that authorized syringe exchange without jurisdictional limitations, removed barriers to possessing and distributing syringes and needles through drug paraphernalia laws, and explicitly allowed for the retail sale of syringes to persons who inject drugs (Figure 2).

**FIGURE 2:**
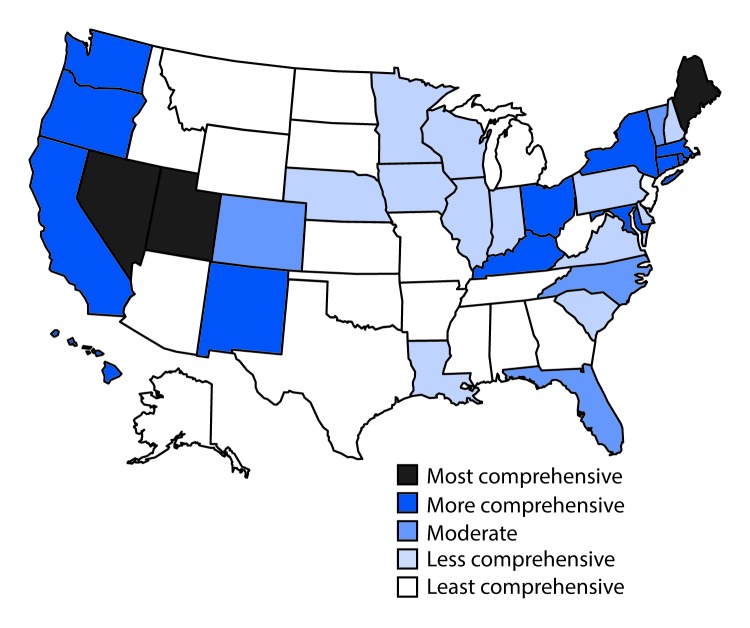
Comprehensiveness* of state laws pertinent to prevention of hepatitis C virus infection among persons who inject drugs — United States, 2016 * Assessment of whether a state had established certain laws and the presence or absence of five elements in those laws, i.e., 1) authorization of syringe exchange statewide or in selected jurisdictions; 2) exemption of needles or syringes from the definition of drug paraphernalia; 3) decriminalization of possession and distribution of syringes or needles for participants of a legally authorized syringe service program; 4) avoidance of criminal prosecution for possession of drug paraphernalia by disclosing possession of a needle or sharp object to an arresting officer; and 5) allowance for the retail sale of syringes without a prescription to persons who inject drugs.

Twenty-four states had restrictive Medicaid treatment policies that required some period of sobriety to receive HCV treatment through Medicaid, including 11 of the states with the least comprehensive set of laws related to prevention. Sixteen states had permissive Medicaid HCV treatment policies that did not require a period of sobriety or only required screening and counseling to receive HCV treatment through Medicaid (Figure 3). Among the seventeen states with high HCV incidence, five (Massachusetts, New Mexico, North Carolina, Pennsylvania, and Washington) had permissive Medicaid treatment policies.

**FIGURE 3:**
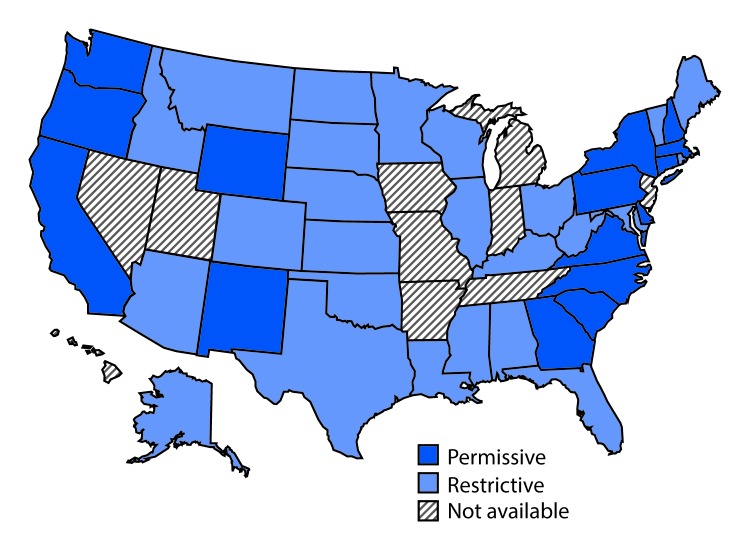
State Medicaid fee-for-service hepatitis C virus treatment policy restrictions* — United States, 2016 * Permissive: Medicaid fee-for-service (FFS) did not require a period of sobriety or only required screening and counseling. Other restrictions, including restrictions based on liver disease or specialty provider requirements are not included; Restrictive: Medicaid FFS required a period of sobriety from drugs and/or alcohol; Not available: No information on Medicaid treatment policy available.

Only three states (Massachusetts, New Mexico, and Washington) had both a most comprehensive or more comprehensive set of laws and a permissive Medicaid treatment policy that might affect access to both HCV preventive and treatment services for persons who inject drugs.

## Discussion

The creation and implementation of law can be used to achieve public health objectives, including infectious disease prevention and control, and legislation can be an effective tool to address public health threats faced by a state’s residents (*9*). To promote HCV prevention, state laws can facilitate access to clean injection equipment, and other services for persons who inject drugs and, thereby be an effective tool to reduce the risk for transmission and stop the increasing incidence of HCV infection in communities, particularly those most affected by the nation’s current opioid epidemic.

The laws governing access to comprehensive HCV prevention services varied in the 17 states with high HCV incidence in 2015. For example among the three states with the highest HCV incidence rates (Kentucky, Massachusetts, and West Virginia), West Virginia had less comprehensive laws, and Kentucky and Massachusetts had more comprehensive laws. However, some of these laws did not take effect until 2015, suggesting that some laws might have been enacted in response to the increased HCV prevalence in these states.

In addition to legal strategies aimed at primary prevention, state Medicaid policies can either facilitate or hinder access to HCV treatment services for persons who inject drugs (*4*). Medicaid treatment policies with strict sobriety requirements can delay or even prevent access to effective and curative treatment (*5*), although access to HCV treatment cures infection, reducing viral transmission and ultimately, incidence, among persons who inject drugs (*10*). Although the costs of HCV therapies have raised budgetary issues for state Medicaid programs in the past, the costs of HCV treatment have declined in recent years, increasing the cost-effectiveness of treatment, particularly among persons who inject drugs and who might serve as an ongoing source of transmission to others (*10*).

The findings in this analysis are subject to at least five limitations. First, the HCV incidence data provided are based on reports of acute HCV cases, representing persons who were recently tested for and received a diagnosis of HCV and were reported to public health authorities. Because most HCV infections are asymptomatic, NNDSS data largely underestimate the prevalence of disease. Furthermore, because HCV reporting requirements and practices differ by state, the degree of underreporting also likely differs by state and should be interpreted with caution. Second, the analysis was conducted at a state level. Local jurisdictions might have implemented different legal or policy interventions that were not captured in this assessment. In addition, this analysis did not consider the enforcement of laws. Third, this cross-sectional, descriptive analysis was based on the most recent surveillance data and the most recent legal data; it is not possible to associate the legal findings with particular incidence rates within states. Additional analysis is needed to understand the impetus behind the laws and to determine their direct impact on HCV incidence, including the impact of case reporting by syringe exchange programs. Fourth, only Medicaid policy data for fee-for-service programs were considered; restrictions in Medicaid managed care programs might differ, other Medicaid barriers to treatment were not assessed, and the direct association between Medicaid sobriety requirements and the number of persons being treated in each state was not assessed. Finally, legal analyses of this nature are largely qualitative, and categorizing states’ policy environments might be subject to reviewer interpretation. However, two separate analysts independently assessed the state laws and Medicaid policies, and their analyses were further validated by state personnel familiar with HCV prevention and treatment activities.

Legal and policy interventions can be tailored to a state’s unique needs to serve as part of a comprehensive strategy for reducing HCV transmission through increased access to preventive services, including safe injection equipment and HCV treatment. The findings from this assessment of state laws and one component of Medicaid treatment policies can inform jurisdictions when building their capacity to prevent the spread of HCV in their communities. Whereas any one policy can have a positive impact on public health, many factors contribute to the prevalence of disease, and it is important for policy makers and public health officials to work together to understand the various needs of particular populations to prevent HCV transmission and disease.

**Summary**

**What is already known about this topic?**

The United States has experienced a sharp increase in hepatitis C virus (HCV) incidence that can be attributed to injection drug use. Some states have used public health laws and treatment policies to reduce the risk for transmission of HCV infections among persons who inject drugs.

**What is added by this report?**

In 2015, seventeen states were characterized as having acute HCV incidence rates above the national average. In an analysis of state laws governing access to safe injection equipment and Medicaid policies related to sobriety requirements for approval of HCV treatment for persons who inject drugs, only three states had state laws and Medicaid policies capable of comprehensively preventing and treating HCV among persons who inject drugs.

**What are the implications for public health practice?**

This report can be used as a tool for states in establishing laws and policies to address increases in HCV incidence in their own jurisdictions, and as a source of data for evaluating the long-term impact of these laws and policies. State laws that increase access to syringe exchange programs and clean needles and syringes, and policies that facilitate access to HCV treatment through state Medicaid programs can reduce HCV transmission risk.

## Footnotes

* http://lawatlas.org/datasets/syringe-policies-laws-regulating-non-retail-distribution-of-drug-parapherna.
