# *Erratum:* Vol. 66, No. 18

**DOI:** 10.15585/mmwr.mm6629a7

**Published:** 2017-07-28

In the report “State HCV Incidence and Policies Related to HCV Preventive and Treatment Services for Persons Who Inject Drugs — United States, 2015–2016,” on page 466, the second sentence of the second paragraph should have read “HCV incidence **rates** increased **by 167%** nationally from **0.3 cases per 100,000 U.S. population in** 2010 to **0.8 in** 2015 (*4*).”

In the cover box “Hepatitis Awareness Month and Testing Day — May 2017,” on page 465, the last sentence of the second paragraph should have read “During 2010–2015, HCV incidence **rates** increased by **167%** with the highest rates among young persons who inject drugs (PWID).^†^”

